# Population pharmacokinetic model of vancomycin in postoperative neurosurgical patients

**DOI:** 10.3389/fphar.2022.1005791

**Published:** 2022-09-26

**Authors:** Shifeng Wei, Dongjie Zhang, Zhigang Zhao, Shenghui Mei

**Affiliations:** ^1^ Department of Pharmacy, Beijing Tiantan Hospital, Capital Medical University, Beijing, China; ^2^ Department of Clinical Pharmacology, College of Pharmaceutical Sciences, Capital Medical University, Beijing, China

**Keywords:** vancomycin, population pharmacokinetic, nonlinear mixed effects modeling, postoperative neurosurgical patients, body weight, estimated glomerular filtration rate, mannitol

## Abstract

**Objective:** Vancomycin is commonly used in postoperative neurosurgical patients for empirical anti-infective treatment due to the low success rate of bacterial culture in cerebrospinal fluid (about 20%) and the high mortality of intracranial infection. At conventional doses, the rate of target achievement for vancomycin trough concentration is low and the pharmacokinetics of vancomycin varies greatly in these patients, which often leads to treatment failure. The objective of this study was to establish a population pharmacokinetic (PPK) model of vancomycin in postoperative neurosurgical patients for precision medicine.

**Method:** A total of 895 vancomycin plasma concentrations from 560 patients (497 postoperative neurosurgical patients) were retrospectively collected. The model was analyzed by nonlinear mixed effects modeling method. One-compartment model and mixed residual model was employed. The influence of covariates on model parameters was tested by forward addition and backward elimination. Goodness-of-fit, bootstrap and visual predictive check were used for model evaluation. Monte Carlo simulations were employed for dosing strategies with AUC_24_ targets 400–600.

**Result:** Estimated glomerular filtration rate (eGFR), body weight (BW) and mannitol had significant influence on vancomycin clearance (CL). $eGFR\left(mL/\min\right)=144\times {\left(Scr/a\right)}^{b}\times 0.993^{age}$ , for female, a = 0.7, Scr ≤ 0.7 mg/dl, b = −0.329, Scr > 0.7 mg/dl, b = −1.209; for male, a = 0.9, Scr ≤ 0.9 mg/dl, b = −0.411, Scr > 0.9 mg/dl, b = −1.210. Vancomycin clearance was accelerated when co-medicated with mannitol and increased with eGFR and BW. In the final model, the population typical value is 7.98 L/h for CL and 60.2 L for apparent distribution volume, $CL\;\left(L/h\right)=7.98\times {\left(eGFR/115.2\right)}^{0.8}\times {\left(BW/70\right)}^{0.3}\times e^{A}$ , where A = 0.13 when co-medicated with mannitol, otherwise A = 0. The model is stable and effective, with good predictability.

**Conclusion:** In postoperative neurosurgical patients, a higher dose of vancomycin may be required due to the augmented renal function and the commonly used mannitol, especially in those with high body weight. Our vancomycin PPK model could be used for individualized treatment in postoperative neurosurgical patients.

## 1 Introduction

Vancomycin is a tricyclic glycopeptide antibacterial drug, which can be used to treat serious infection caused by Gram-positive bacteria especially for methicillin-resistant *staphylococcus aureus* (Reuter et al., 2022). Vancomycin has a clear dose-response and dose-toxicity relationship with narrow therapeutic window and large pharmacokinetic variations between individuals (Vandecasteele et al., 2013). The ratio of area under the concentration-time curve (AUC_24_) to minimal inhibit concentration (MIC) of 400–600 is a recognized pharmacokinetic/pharmacodynamic index for vancomycin in clinical practice, while high AUC_24_ (>650) significantly increases its nephrotoxicity (Aljefri et al., 2019; Murphy et al., 2020; Rybak et al., 2020; Reuter et al., 2022). Vancomycin guideline recommend that the serum vancomycin trough concentration in adult patients with *S. aureus* infection should be within 15–20 mg/L (Rybak et al., 2020). Vancomycin trough concentration ≥15 mg/L significantly reduced the rate of treatment failure in adult bacteremia patients, while the risk of nephrotoxicity was significantly increased with the vancomycin trough concentration (Murphy et al., 2020; Tsutsuura et al., 2021; Reuter et al., 2022).

Vancomycin is almost not absorbed by oral administration and needs to be administered intravenously. The protein binding rate is about 10%–50%, and more than 80% of vancomycin is excreted in prototype through the kidney within 24 h after administration (Rybak, 2006). In patients with normal renal function, vancomycin has an *α* half-life of approximately 0.5–1 h and a terminal elimination half-life of 3–9 h (Rybak, 2006). Vancomycin has complex pharmacokinetic characteristics with large inter-individual variations. Vancomycin population pharmacokinetics (PPK) model can quantitatively explain the influence of various factors on pharmacokinetic parameters, which is helpful for precision medicine (Lin et al., 2021). Studies have shown that renal function and body weight (BW) were the most important influencing factors for vancomycin clearance (CL) and apparent volume of distribution (V), respectively, and both two factors should be considered for its dose adjustment (Lin et al., 2021; Revilla et al., 2010; Medellín-Garibay et al., 2017; Mangin et al., 2014; Llopis-Salvia and Jiménez-Torres, 2006; Jalusic et al., 2021; Yang et al., 2017; Huang et al., 2021; del Mar Fernández de Gatta Garcia et al., 2007; CHEN et al., 2013; Marsot et al., 2012; Colin et al., 2019). Furthermore, sex, age, serum albumin levels, disease status, concomitant drugs (e.g., meropenem and dopamine), and mechanical support (e.g., renal replacement therapy (RRT) and mechanical ventilation) could also affect vancomycin pharmacokinetics (Lin et al., 2021; Revilla et al., 2010; Medellín-Garibay et al., 2017; Mangin et al., 2014; Jalusic et al., 2021; Yang et al., 2017; del Mar Fernández de Gatta Garcia et al., 2007; Yang et al., 2018; Bang et al., 2022; ZENG et al., 2016; Alqahtani et al., 2018; Economou et al., 2018; Garreau et al., 2021; Jing et al., 2020; Kanji et al., 2020; Wang et al., 2021; Kim et al., 2016; Li et al., 2017; Zhang et al., 2020).

Vancomycin is commonly used in postoperative neurosurgical patients for empirical anti-infective treatment due to the low success rate of bacterial culture in cerebrospinal fluid (about 20%) and the high mortality of intracranial infection (Tunkel et al., 2017; Hasbun, 2021; Schneider et al., 2022). Postoperative neurosurgical patients usually experience significant pathophysiological changes, including the destruction of the blood-brain barrier or blood-cerebrospinal fluid barrier, the increase of heart output, and augmented renal function (Blot et al., 2014; Xiao et al., 2022). Previous studies have shown that the CL of vancomycin in postoperative neurosurgical patients was significantly increased (Kim et al., 2016; Lin et al., 2016; Li et al., 2017; Jing et al., 2020; Jalusic et al., 2021). The rate of target achievement for vancomycin trough concentration is low under conventional dose in postoperative neurosurgical patients, which often leads to treatment failure (Morbitzer et al., 2019; Chen et al., 2020). However, little is known about the factors influencing the pharmacokinetics of vancomycin in these patients. Therefore, it is quite necessary to establish a PPK model of vancomycin in postoperative neurosurgical patients for personalized medicine.

## 2 Materials and methods

### 2.1 Study design

This study was in accordance with the Helsinki Declaration and approved by the Ethics Committee of Beijing Tiantan Hospital, Capital Medical University, Beijing, China (ID: KY 2022-01802).

The data of patients who received vancomycin therapy from October 2018 to March 2022 in Beijing Tiantan Hospital, Capital Medical University were retrospectively collected. Inclusion criteria were: 1) age ≥18 years; 2) therapeutic drug monitoring was performed during treatment and at least 1 vancomycin concentration was obtained. Exclusion criteria were: 1) pregnant women (Lin et al., 2021); 2) cystic fibrosis (Jing et al., 2020).

The following information was extracted: 1) demographic characteristics (sex, age, height, BW, and body mass index); 2) dosing information of vancomycin (date, time, dose, sampling time, and serum concentration); 3) concomitant drugs (mannitol, meropenem and diuretics such as furosemide or torasemide); 4) renal function and the use of RRT.

The dosage regimen of vancomycin is adjusted by the clinicians according to the patient’s conditions and renal function, including 1 g Qd, 1 g Q12h, 1 g Q8h, 0.5 g Qd, 0.5 g Q12h, 0.5 g Q8h and 0.5 g Q6h. The infusion time cannot be accurately obtained, but 1 h infusion was most used in clinical applications, so the default infusion time in this study was 1 h. Vancomycin serum concentrations were obtained from patient’s therapeutic drug monitoring data, most of which were steady state (>4 doses) trough concentrations. The blood samples were anticoagulated by Ethylene Diamine Tetraacetic Acid, centrifuged at 5,000 rpm for 3 min and analyzed by chemiluminescence immunoassay (ADVIA Centaur^®^ XP, Siemens). Sample calibration and quality control were carried out in accordance with the manufacturer’s instructions and the calibration range was 0.67–90 mg/L.

### 2.2 Development of population pharmacokinetic model

In this study, nonlinear mixed effects modeling (NLME) method was used to construct the PPK model. Pharmacokinetic parameters and the variability were assessed using the first-order conditional estimation with extended least square method (FOCE-ELS) by using the Phoenix^®^ NLME software (version 8.3; Certara, St. Louis, Missouri). Bootstrap and visual predictive check (VPC) were used to test the stability and predictive ability of the final model.

#### 2.2.1 Base model

The one-compartment model with first-order elimination was used to describe the pharmacokinetic characteristics of vancomycin. The sparse trough concentration data led to the inaccurate estimation of the V. Therefore, the population typical value of V was fixed at 60.2 L according to a PPK model in Chinese neurosurgery adult patients (Jing et al., 2020).

Inter-individual variability (*η*) of PPK model was described by exponential random effect model in Eq. 1. Residual variability (*ε*) was described by combined (multiplicative and additive) error model in Eq. 2. The *η* was assumed to follow a normal distribution with a mean of zero and a variance of *ω* ^2^. The *ε* was assumed to follow a normal distribution with a mean of zero and a variance of *σ* ^2^.

$$\theta_{i}=\theta_{TV}\times e^{\eta i},$$ (1)

$$C_{obs}=C_{pred}+\varepsilon \times \sqrt{1+{\left(C_{pred}\times \sigma_{1}/\sigma_{2}\right)}^{2}}$$ (2)

where *θ*_*TV*_ is the population typical value of pharmacokinetic parameters, *θ*_*i*_ is the individual (*i* ^th^) pharmacokinetic parameters. Cobs and Cpred respectively represent the measured concentration and predicted concentration of a patient; *σ*_1_ represents the multiplicative *σ*, and *σ*_2_ represents the standard deviation of *ε*.

#### 2.2.2 Covariate model

Continuous covariates included age, height, BW, body mass index, serum creatinine (Scr), creatinine clearance (CLcr) and estimated glomerular filtration rate (eGFR). Categorical covariates included sex, RRT and concomitant drugs (mannitol, meropenem and diuretics). CLcr is calculated by the Cockcroft-Gault equation (Cockcroft and Gault, 1976). eGFR is calculated by the Chronic Kidney Disease Epidemiology Collaboration (CKD-EPI) equation (Kong et al., 2013). All continuous covariates are centered at their median values. The difference of the objective function value (OFV) was used to compare the models.

Cockcroft-Gault equation:

$$CLcr\left(mL/\min\right)=\frac{\left(140-age\left(year\right)\right)\times BW\left(kg\right)}{Scr\left(mg/dL\right)\times 72}\;\left(\times 0.85\;if\;female\right).$$ (3)

CKD-EPI equation:

$$eGFR\left(mL/\min\right)=144\times {\left(\frac{Scr}{a}\right)}^{b}\times 0.993^{age}.$$ (4)

For female, a = 0.7, Scr ≤ 0.7 mg/dl, b = –0.329, Scr > 0.7 mg/dl, b = −1.209; for male, a = 0.9, Scr ≤ 0.9 mg/dl, b = −0.411, Scr > 0.9 mg/dl, b = −1.210.

After the establishment of the base model, the effects of covariates on pharmacokinetic parameters were tested by forward addition and backward elimination under stepwise manner. In the forward addition, if the decrease of OFV was greater than 6.64 (*p* < 0.01, df = 1), the covariate was considered to have a significant influence on the model parameters. In the backward elimination, if the increase of OFV was greater than 10.83 (*p* < 0.001, df = 1), the covariate was considered to have a significant impact on the model parameters, and it was retained in the model, otherwise it was removed from the model.

#### 2.2.3 Goodness-of-fit and model evaluation

The OFV values and scatter plots were used to evaluate the goodness-of-fit between the base model and the final model. The model fitting was evaluated by observing the consistency between the observed concentration and population predicted concentration (PRED), and the distribution uniformity of conditional weighted residuals (CWRES) versus PRED or time (Lin et al., 2016). Bootstrap and VPC were performed to evaluate the stability and predictive ability of the final model. For bootstrap, 5,000 data sets were generated by repeatedly sampling the original data sets. The model parameters of each data set were calculated and recorded. The median and 95% confidence interval (2.5%–97.5%) for the estimated parameters were calculated and compared with the final model estimation (Lin et al., 2016). For VPC, 10,000 virtual data sets were generated using the final PPK model, and the 5th, 50th, and 95th percentile of simulated results (90% prediction interval) were calculated to compare with the distribution of observed values (Li et al., 2017).

#### 2.2.4 Simulations for dose selection

Monte Carlo simulation was performed to predict vancomycin concentrations in patients with typical characteristics using different dosing regimens. The default dosing interval was 12 h and the infusion time was 1 h for all simulations. Each regimen was simulated 1,000 times. The AUC_24_ within 400–600 h mg/L is pharmacokinetic/pharmacodynamic index for vancomycin (Rybak et al., 2020), therefore, the recommended dose regimen to achieve the pharmacokinetic/pharmacodynamic index was given out for each type of patients.

## 3 Results

### 3.1 Characteristics of study patients

A total of 895 serum vancomycin concentrations collected from 560 (497 postoperative neurosurgical patients) Chinese adult patients were used for PPK model development. The most common neurological disorders were intracranial space-occupying lesions. Demographic characteristics, renal function and concomitant medications of patients are summarized in Table 1. The histogram plots of the covariates are shown in Supplementary Appendix S1. Vancomycin concentration was 14.20 ± 7.36 mg/L, and only 20.4% (183/895) reached the target trough concentration of 15–20 mg/L. In our study, 2.72% of patients had severe renal insufficiency and most of these patients were treated with RRT during vancomycin therapy; 71.32% of patients were co-medicated with meropenem, 15.95% of patients used diuretics to relieve cerebral edema, and 60.32% of patients used mannitol to relieve cerebral edema and to reduce intracranial pressure.

**TABLE 1:** Characteristics of patients in the population pharmacokinetic model of vancomycin.

Variable	Mean ± standard deviation (minimum to maximum)
NO. of. Subjects/Observation	560/895
Sex (Male/Female)	370/190
Age (years)	52.41 ± 15.11 (18–89)
Body weight (kg)	69.74 ± 13.05 (37.5–130)
Height (cm)	167.88 ± 7.98 (145–192)
Body mass index (kg/m^2^)	24.64 ± 3.64 (15.61–47.75)
Dose (mg/time)	951.19 ± 152.23 (50–1500)
Concentration (mg/L)	14.20 ± 7.36 (0.91–52.96)
Serum creatinine (μmol/L)	64.87 ± 76.89 (9.79–957.5)
creatinine clearance (ml/min)	152.94 ± 74.89 (5.98–903.23)
eGFR (ml/min)	112.74 ± 30.91 (3.52–244.48)
Renal replacement therapy (%)	2.72%
Concomitant drugs (used, %)	
Meropenem	71.32%
Mannitol	60.32%
Diuretics	15.95%

### 3.2 Development of population pharmacokinetic model

In the base model, additive, multiplicative, exponential and combined (multiplicative and additive) error models were tested for residual variability, and the combined error model was used. After the development of the base model, covariates selection results are shown in Table 2. Compared with the base model, eGFR was the most significant covariate on vancomycin clearance (ΔOFV = −626.80). In the final PPK model, eGFR, mannitol and BW had significant influence on vancomycin clearance.

**TABLE 2:** Results in the model development procedure of vancomycin population pharmacokinetic model.

Model No.	Model description	OFV	∆OFV	*p* value
Forward addition				
1	Base model	6147.4050		
2	Add eGFR on CL in model 1	5520.6043	−626.8007	<0.01
3	Add CLcr on CL in model 1	5589.9172	−557.4878	<0.01
4	Add Scr on CL in model 1	5750.6547	−396.7503	<0.01
5	Add RRT on CL in model 1	5904.7012	−242.7038	<0.01
6	Add age on CL in model 1	6070.1077	−77.2973	<0.01
7	Add mannitol on CL in model 1	6109.9026	−37.5024	<0.01
8	Add BW on CL in model 1	6136.6218	−10.7832	<0.01
9	Add height on CL in model 1	6138.3224	−9.0826	<0.01
10	Add diuretics on CL in model 1	6140.9679	−6.4371	>0.01
11	Add body mass index on CL in model 1	6142.5322	−4.8728	>0.01
12	Add meropenem on CL in model 1	6147.2899	−0.1151	>0.01
13	Add sex on CL in model 1	6147.3567	−0.0483	>0.01
14	Add mannitol on CL in model 2	5488.8230	−31.7813	<0.01
15	Add BW on CL in model 14	5464.6719	−24.1511	<0.01
Backward elimination				
16	Remove eGFR on CL from model 15	6098.0772	633.4053	<0.001
17	Remove mannitol on CL from model 15	5498.2604	33.5885	<0.001
18	Remove BW on CL from model 15	5488.8230	24.1511	<0.001

OFV, objective function value; CL, clearance rate; eGFR, estimated Glomerular Filtration Rate; CLcr, creatinine clearance; Scr, serum creatinine; RRT, renal replacement therapy; BW, body weight.

The final model shows that the typical value of CL is 7.98 L/h. Compared with the base model, the random inter-individual variability of CL in the final model was significantly reduced (21.45% vs. 48.19%). Standard errors of all parameters (1.90%–25.83%) in our model are acceptable. The parameter estimates, relative standard error, 95% confidence intervals, inter-individual variability and intra-individual residual variability of the base model and final model, and bootstrap results are shown in Table 3. The quantitative relationships between model parameters and covariates are listed below:

$$CL\;\left(L/h\right)=7.98\times {\left(eGFR/115.2\right)}^{0.8}\times {\left(BW/70\right)}^{0.3}\times e^{A},$$ (5)

V(L)=60.2 (6)

**TABLE 3:** Parameter estimates and Bootstrap results of vancomycin population pharmacokinetic model.

Parameters	Base model		Final model		Bootstrap	
	Estimate (%RSE)	95% CI	Estimate (%RSE)	95% CI	Median (%RSE)	95% CI
V (L)	60.2	—	60.2	—	60.2	—
CL (L/h)	8.08 (1.93)	7.74–8.36	7.98 (1.90)	7.68–8.28	7.97 (2.24)	7.62–8.32
eGFR on CL (L/h)	—	—	0.80 (4.30)	0.74–0.87	0.80 (4.39)	0.74–0.88
Mannitol on CL (L/h)	—	—	0.13 (17.85)	0.08–0.17	0.13 (17.72)	0.08–0.17
BW on CL (L/h)	—	—	0.30 (20.19)	0.18–0.42	0.30 (19.91)	0.18–0.42
IIV_CL_ (CV%)	48.19	—	21.45	—	21.23	—
σ_1_ (multiplicative, CV)	0.19 (13.06)	0.14–0.24	0.25 (6.45)	0.22–0.28	0.25 (7.82)	0.20–0.28
σ_2_ (additive, mg/L)	2.73 (13.69)	1.99–3.46	1.51 (25.83)	0.75–2.28	1.50 (29.10)	0.80–2.48

V, volume of distribution; CL, clearance rate; eGFR, estimated Glomerular Filtration Rate; BW, body weight; IIV, inter-individual variability; σ, residual variability.

When co-medicated with mannitol, A = 0.13; otherwise, A = 0.

### 3.3 Goodness-of-fit and model evaluation

**FIGURE 1:**
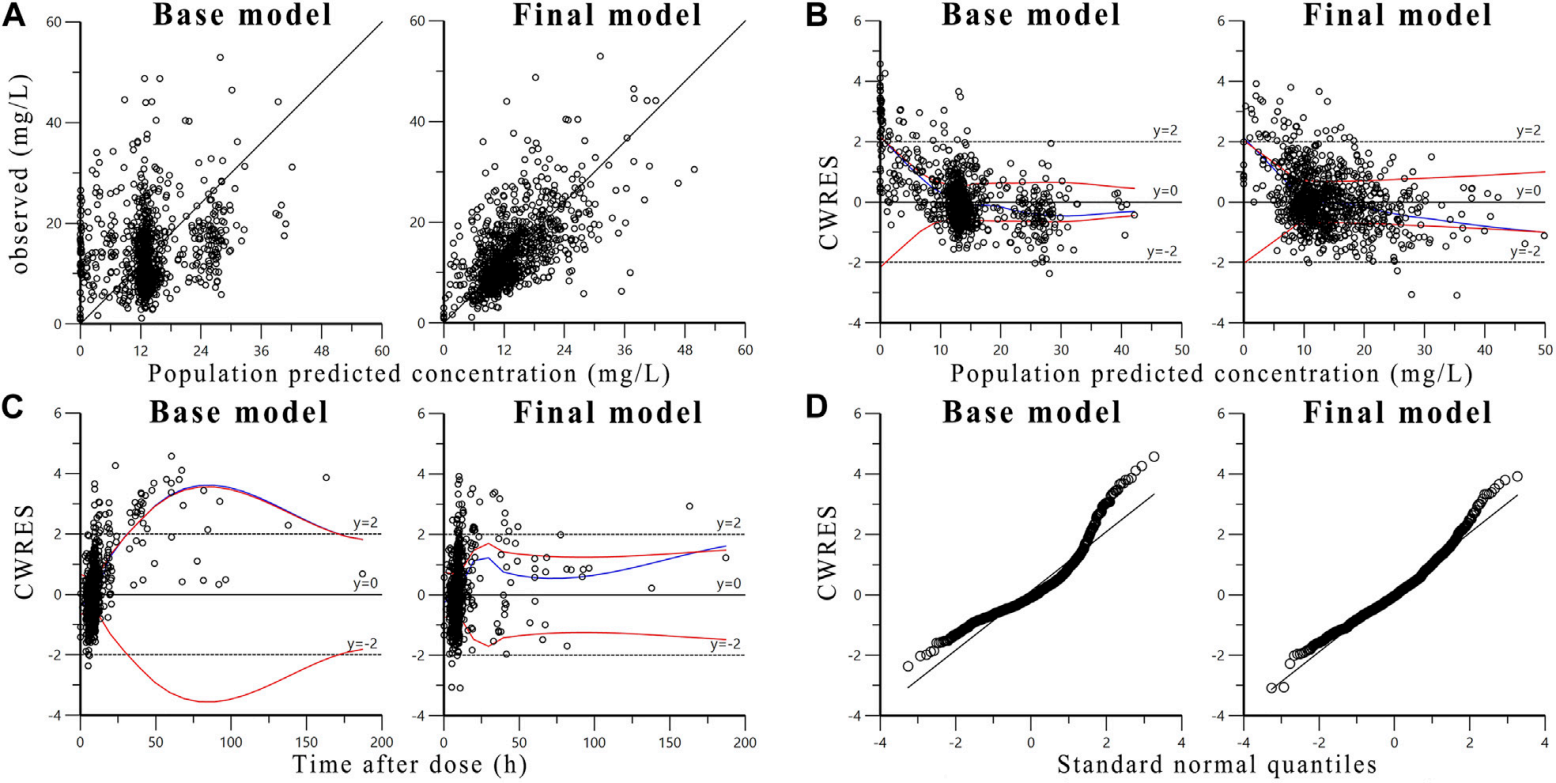
Diagnostic goodness-of-fit plots of base model and final model: **(A)** The observed concentrations versus population predicted concentrations; **(B)** The conditional weighted residuals (CWRES) versus population predicted concentrations; **(C)** The conditional weighted residuals versus time after dose; **(D)** The conditional weighted residuals versus standard normal quantiles. The blue line is the overall trend of data fitting, the two red lines are the absolute value distribution of the data and its mirror image, respectively.

Diagnostic plots of base model and final model is shown in Figure 1 to evaluate the goodness-of-fit of the final model. The scatter plot of observed concentration versus PRED (Figure 1A) shows that the final model significantly improves data fitting compared to the base model. The population prediction values of the final model are close to the observed values and are randomly and uniformly distributed on both sides of the curve, with small variation (Lin et al., 2021). The scatter plots of CWRES versus PRED (Figure 1B) and CWRES versus time after dose (Figure 1C) show that there is no obvious bias between the PRED, time after dose and CWRES. The prediction errors of most concentration points are within two times of the standard deviation, evenly distributed on both sides of the coordinate axis, and do not change with concentration and time (ZENG et al., 2016). These results indicating that the model fitting is good without obvious deviation. The quantile-quantile plots of CWRES (Figure 1D) indicate that, the η and ε of the final model are close to normal distribution, which are consistent with the modeling assumptions.

The bootstrap results are shown in Table 3. The median and distribution of parameters obtained by bootstrap are close to the estimated values of the final PPK model. The parameter values of the final model are all within the 95% confidence intervals of the corresponding parameter values of bootstrap, indicating that the final model is stable (Lin et al., 2016). The VPC results of the final PPK model are shown in Supplementary Appendix S2. In general, 95.3% (853/895) of observed vancomycin concentrations fall inside the 90% prediction interval, indicating that the final model has a good predictive ability (Kim et al., 2016).

### 3.4 Simulations for dose selection

The simulated dose, AUC_24_ and its 90% confidence interval (5%–95%) by the final model for different type of patients are listed in Table 4. For example, for a patient with body weight of 65 kg and eGFR of 90 ml/min and co-medicated with mannitol, the recommended dose regimen would be 1,450 mg Q12h (AUC_24_ = 400 h mg/L)−2,150 mg Q12h (AUC_24_ = 600 h mg/L). In addition, it should be noted that when the daily dose of intravenous vancomycin is too high, a low-dose intraventricular administration regimen (10–20 mg every 24 h) is recommended to prevent drug toxicity (Nau et al., 2020).

**TABLE 4:** The simulated dose regimen in patients with typical characteristics by the final model.

eGFR (ml/min)	Body weight (kg)	Dosing interval (h)	Mannitol = yes				Mannitol = no			
			Dose 1 (mg)	AUC_24_ (400) (90% CI)	Dose 2 (mg)	AUC_24_ (600) (90% CI)	Dose 1 (mg)	AUC_24_ (400) (90% CI)	Dose 2 (mg)	AUC_24_ (600) (90% CI)
90	85	12	1600	401.47 (293.80–574.70)	2350^a^	600.50 (414.31–844.34)	1400	402.19 (275.46–577.10)	2050^a^	593.86 (409.72–834.99)
90	65	12	1450	400.46 (278.89–563.07)	2150^a^	596.63 (425.29–864.34)	1300	405.62 (290.79–567.37)	1900	598.44 (424.16–877.44)
45	85	12	900	402.28 (283.58–564.73)	1350	600.04 (412.90–858.12)	800	401.47 (286.27–587.27)	1200	607.06 (429.45–854.37)
45	65	12	825	398.85 (275.21–560.15)	1250	607.36 (425.11–848.61)	750	406.38 (283.88–582.65)	1100	600.75 (421.69–847.62)
15	85	12	375	399.33 (279.04–541.50)	560	596.34 (416.70–808.63)	330	394.54 (277.72–528.21)	500	593.28 (421.08–792.91)
15	65	12	350	399.18 (186.48–546.08)	525	602.02 (422.52–809.92)	310	398.11 (281.88–528.18)	465	597.17 (422.82–792.28)

a Switching from the high-dose intravenous regimen to the low-dose intraventricular regimen (10–20 mg every 24 h) is recommended.

## 4 Discussion

### 4.1 Model development and validation

One, two and three compartment models have been used for model fitting in published vancomycin PPK studies in critically ill patients. Studies showed that the pharmacokinetic characteristics of vancomycin in adult patients could be typically described by a two-compartment model (Marsot et al., 2012; Colin et al., 2019). As a retrospective study, most of the samples were trough concentrations. The lack of data at distribution phase resulted in the inaccurate estimation of the parameters for the two-compartment model (Jing et al., 2020). Previous studies showed that estimation of vancomycin pharmacokinetic parameters (clearance and covariate effects) with one-compartment model was reliable (Lin et al., 2016; Zeng et al., 2016; Yang et al., 2018; Jing et al., 2020; Zhang et al., 2020; Lin et al., 2021). Therefore, one-compartment model was used for data fitting in this study.

The random effects errors in our model were acceptable. The interindividual variability of CL (21.45%) was small, compared to other vancomycin models in neurosurgical patients (21%–61.92%) (Li et al., 2015; Kim et al., 2016; Lin et al., 2016; Li et al., 2017; Ding et al., 2018; Jing et al., 2020; Jalusic et al., 2021). The residual variability (19%, 2.73 mg/L) was close to that (18.5%, 3.16 mg/L) of the model developed by Jalusic et al. (2021). The η-shrinkage of CL and ε-shrinkage in the final model were 0.20 and 0.23, respectively, indicating that the individual predictions are valuable for assessing model adequacy.

The weakness of trough level observations resulted in the inaccurate estimation of V. Therefore, the V was fixed at 60.2 L according to a PPK model based on the same race (Chinese), disease type (post-neurosurgery), compartment model (one-compartment model) and patient characteristics (age, body weight, etc.) (Jing et al., 2020). The V of 60.2 L was close to the value (54 ± 17 L) in another eligible study (Lin Wu et al., 2015).

The published studies have demonstrated a significant increase in vancomycin clearance (0.10–0.13 L/h/kg) in neurosurgical patients compared to other patient groups (0.031–0.086 L/h/kg) [(Marsot et al., 2012; Kim et al., 2016; Lin et al., 2016; Li et al., 2017; Jing et al., 2020; Jalusic et al., 2021), (Li et al., 2015; Lin Wu et al., 2015; Ding et al., 2018; Chen et al., 2022)]. In this study, the typical value of vancomycin CL (0.11 L/h/kg) was high. Kassel et al. (2018) reported that, in adult neurologically critically ill patients, compared with the 1 g Q12h group, the 1 g Q8h group was more likely to reach the target trough concentrations (>15 mg/L) at initial measurement and at 7–10 days, and was easier to achieve the pharmacodynamic target when MIC was high. This need for dose increase might be secondary to the augmented renal clearance (Kassel et al., 2018), which was a common phenomenon in critically ill patients characterized by increased creatinine clearance (CLcr greater than or equal to 130 ml/min/1.73 m^2^) (Xiao et al., 2022). The mechanism of augmented renal clearance was not fully elucidated, and it was related to enhanced metabolism, altered neurohormonal balance, and fluid resuscitation (Cook and Hatton-Kolpek, 2019). Young (<50 years), male, severe neurological injury, sepsis, trauma, and burns might be risk factors for augmented renal clearance (Mahmoud and Shen, 2017). Autonomic dysfunction and paroxysmal sympathetic hyperactivity will occur after traumatic brain injury (Khalid et al., 2019). The increase in atrial natriuretic peptide may play a role in augmented renal clearance, which can be explained by the increased myocardial contractility due to increased sympathetic activity (Khalid et al., 2019). In addition, augmented renal clearance is associated with increased cardiac output, suggesting that augmented cardiovascular function is a contributing factor to augmented renal clearance (Udy et al., 2010; Khalid et al., 2019). 68.2% (382/560) of our patients experienced augmented renal clearance during therapeutic drug monitoring, which explained the high vancomycin clearance in our study.

The Cockcroft-Gault equation is the most commonly used equation for CLcr calculation for its simplicity and convenience (Cockcroft and Gault, 1976). The CKD-EPI equation shows good efficiency for eGFR estimation in Chinese population (Kong et al., 2013). Xie et al. (2013) analyzed the ability of five equations (CKD-EPI, 24hScr, Cockcroft-Gault, Modification of Diet in Renal Disease equation and its modified equation for Chinese population) for eGFR estimation in Chinese patients with chronic kidney disease, and they found that the CKD-EPI equation was the best to estimate eGFR in these patients. The Cockcroft-Gault equation and the CKD-EPI equation were used to calculate CLcr and eGFR, both of which were used to represent the renal function of our patients.

In published PPK studies of vancomycin, CLcr, eGFR and Scr were used to adjust vancomycin CL, which could be explained by the fact that vancomycin was mainly excreted through the kidney (Revilla et al., 2010; Mangin et al., 2014; Lin et al., 2016; Li et al., 2017; Alqahtani et al., 2018; Jing et al., 2020; Kovacevic et al., 2020; Zhang et al., 2020; Garreau et al., 2021; Huang et al., 2021; Jalusic et al., 2021). CLcr, eGFR and Scr were separately used to adjust vancomycin clearance for model construction. The data fitting of the final CLcr model (OFV = 5510.73) and Scr model (OFV = 5536.97) was worse than that of eGFR model (OFV = 5464.67), indicating that eGFR was the best indicator for the adjustment of vancomycin CL in our patients.

Vancomycin clearance was increased with BW in the final model, which was consistent with the results in the literature (Mangin et al., 2014; Jing et al., 2020; Lin et al., 2021; Wang et al., 2021; Bang et al., 2022). The influence of BW on CL is related to various physiological changes, including increased renal mass and cardiac output, both of which could increase renal blood flow, and ultimately increased vancomycin CL (Grace, 2012).

Concomitant medications could also affect vancomycin pharmacokinetic parameters in critically ill patients, meropenem and dopamine could increase vancomycin CL (YANG et al., 2018; Lin et al., 2021). In this study, we tested the effect of common concomitant medications (meropenem, mannitol and diuretics) on vancomycin CL. Co-medicated with mannitol resulted in a 14% increase in vancomycin CL, but the influence of meropenem on vancomycin CL was not observed. Mannitol is a hemodynamically active agent and hypertonic diuretic widely used to reduce intracranial pressure, which may alter renal blood flow, thereby enhancing renal clearance (Lin et al., 2016). Chen et al. (2021) found that postoperative neurosurgical patients co-medicated with mannitol or furosemide had significantly lower vancomycin concentrations than those without using these drugs (*p* = 0.004), which was consistent with our findings.

We summarized the PPK models of vancomycin in critically ill patients. The use of RRT, different RRT techniques and intensities affected vancomycin CL (Economou et al., 2018; Kanji et al., 2020; Garreau et al., 2021; Lin et al., 2021; Wang et al., 2021). Vancomycin CL was reduced in patients receiving RRT compared with those without RRT (Lin et al., 2021). Wang et al. (2021) reported that vancomycin CL was increased with the ultrafiltration rate in critically ill patients. In this study, the influence of RRT on vancomycin CL was not observed, because both effects of RRT and eGFR on CL could be explained by the influence of renal function on vancomycin excretion. There was a strong correlation between RRT and eGFR in our patients, the effect of RRT on CL was covered by the effect of eGFR on CL. On the other hand, the sparse data of RRT may hinder to estimate its effect on vancomycin CL.

Other factors could also affect vancomycin CL. Vancomycin CL inversely related to albumin levels, which could affect the concentration of free drug (Wang et al., 2021). Interestingly, the infusion of albumin could accelerate the conversion rate of vancomycin from the central compartment to the peripheral compartment but did not affect vancomycin CL (Yang et al., 2017). Vancomycin CL was reduced in patients receiving mechanical ventilation, which might be due to changes in hemodynamics (lower cardiac output, lower renal blood flow, decreased eGFR and urine flow) and protein permeability of alveolar capillary membrane (Medellín-Garibay et al., 2017). Moreover, vancomycin CL decreased with the deterioration of the disease, indicators related to the severity of patients’ disease (the Simplified Acute Physiological Score and the Acute Physiology and Chronic Health Evaluation) could also influence vancomycin CL (Mangin et al., 2014; ).

### 4.2 Deficiencies of the study

1) Lack of data at distribution phase in our retrospective study resulted in imprecise estimation of the V value of the model, which was fixed at 60.2 L.
2) The protein binding rate varies between different patient populations (Oyaert et al., 2015). The vancomycin concentration was the total drug concentration, and the unbound concentration was unknown in our study.
3) Clinicians should do their best to avoid using nephrotoxic drugs in patients treated with vancomycin, but other co-medicated drugs that might have effects on renal function were not considered in this study.
4) The effect of dopamine on pharmacokinetic parameters of vancomycin was not verified.
5) Due to the lack of relevant data, the effect of serum albumin level, disease severity and the presence of mechanical ventilation on vancomycin CL were not estimated.
6) External validation was not performed.

## 5 Conclusion

A vancomycin PPK model was successfully established in postoperative neurosurgical patients. eGFR, BW and mannitol combination had significant effects on vancomycin CL. The population typical value of CL in the final model is 7.98 L/h, $CL\;\left(L/h\right)=7.98\times {\left(eGFR/115.2\right)}^{0.8}\times {\left(BW/70\right)}^{0.3}\times e^{A}$ , A = 0.13 when co-medicated with mannitol, otherwise A = 0. The final PPK model is stable and reliable after validation. Higher doses of vancomycin were required to achieve therapeutic effect in postoperative neurosurgical patients due to the increased vancomycin CL caused by augmented renal clearance, and commonly co-medicated with mannitol especially in patients with high body weight. This model might be useful for individualized therapy of vancomycin in postoperative neurosurgical patients.

## Data Availability

The raw data supporting the conclusion of this article will be made available by the authors, without undue reservation.

## Supplementary material

SUPPLEMENTARY APPENDIX S1 The histogram plots of the covariates.

SUPPLEMENTARY APPENDIX S2 Visual predictive check results of final model: **(A)** Patients not co-medicated with mannitol; **(B)** Patients co-medicated with mannitol.

## Abbreviations

AUC, area under concentration-time curve; MIC, minimal inhibit concentration; PPK, population pharmacokinetic; BW, body weight; CL, clearance; V, apparent volume of distribution; RRT, renal replacement therapy; VPC, visual predictive check; Scr, serum creatinine; CLcr, creatinine clearance; eGFR, estimated glomerular filtration rate; CKD-EPI, chronic kidney disease Epidemiology Collaboration; OFV, objective function value; PRED, population predicted concentration; CWRES, conditional weighted residuals.
