# Supplementary figures and images for: Population pharmacokinetic model of vancomycin in postoperative neurosurgical patients
Source: Front Pharmacol. 2022 Sep 26;13:1005791. doi: 10.3389/fphar.2022.1005791 (PMC9548544; doi:10.3389/fphar.2022.1005791)

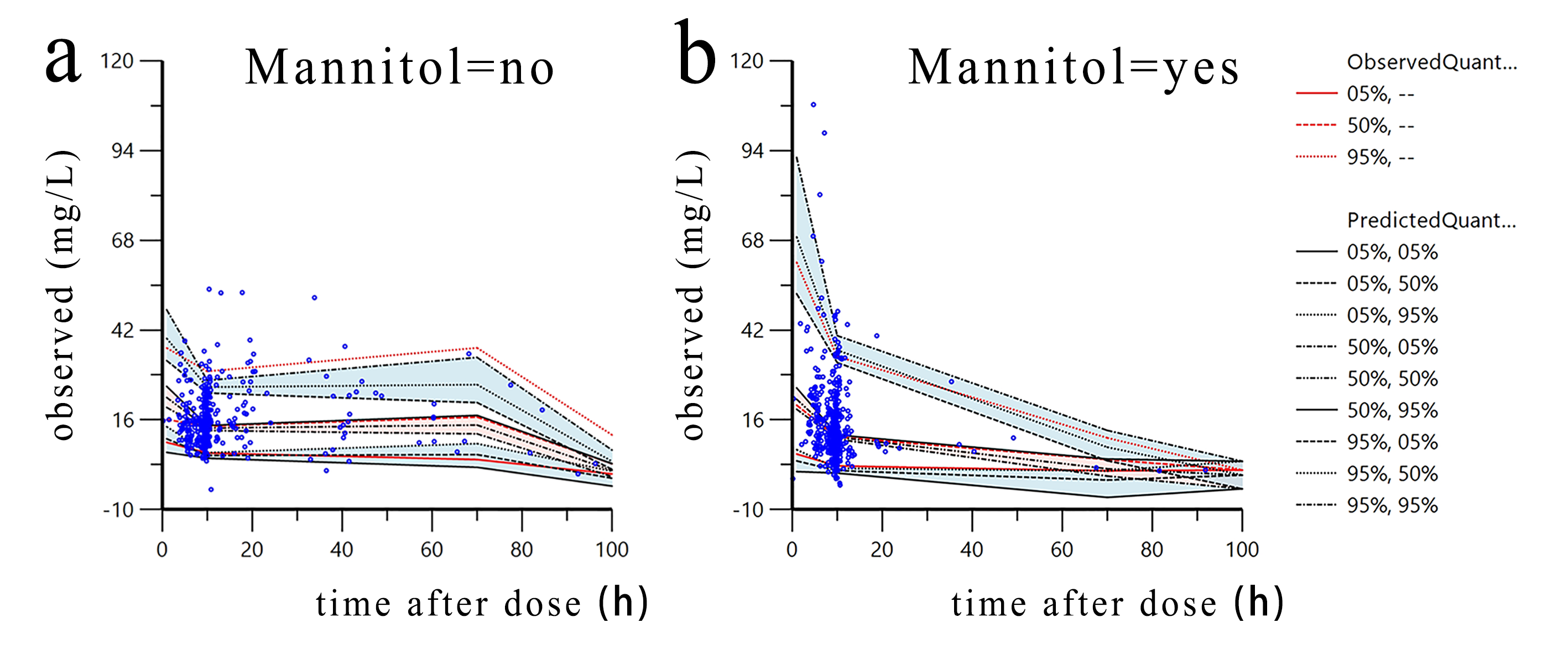

Supplement: Supplementary file 1 [file Image2.TIF]

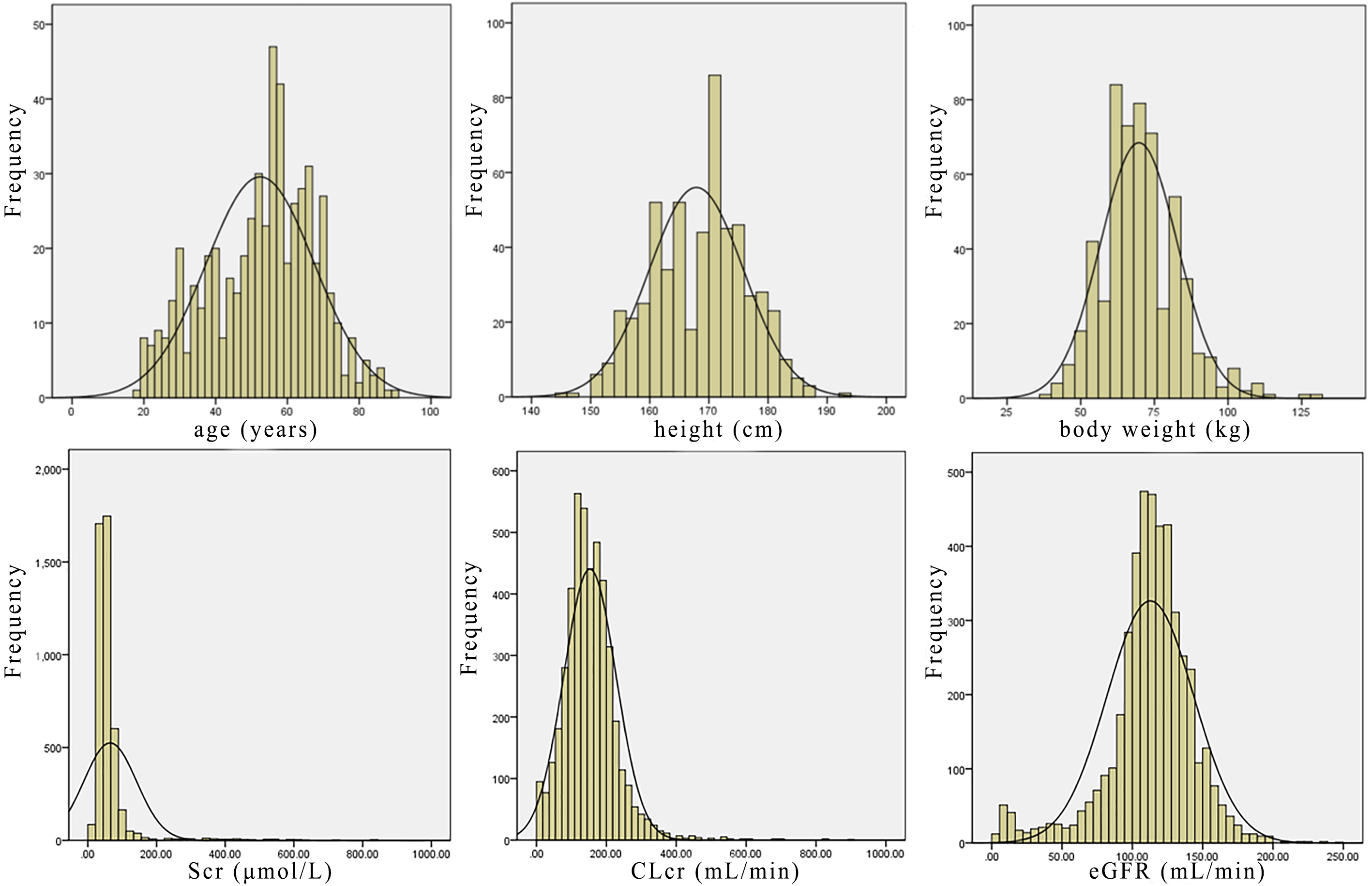

Supplement: Supplementary file 2 [file Image1.TIF]
